# Analysis of coal face stability of lower coal seam under repeated mining in close coal seams group

**DOI:** 10.1038/s41598-021-04410-5

**Published:** 2022-01-11

**Authors:** Yu Xiong, Dezhong Kong, Zhijie Wen, Guiyi Wu, Qinzhi Liu

**Affiliations:** 1 College of Mining, Guizhou University, Guiyang, 550025 China; 2 Key Laboratory of Mining Disaster Prevention and Control, Qingdao, 266590 China; 3 College of Energy and Mining Engineering, Shandong University of Science and Technology, Qingdao, 266590 China

**Keywords:** Geology, Petrology, Coal

## Abstract

Aiming at the problem of coal face failure of lower coal seam under the influence of repeated mining in close coal seams, with the working face 17,101 as a background, the coal samples mechanics test clarified the strength characteristics of the coal face under repeated mining, through similar simulation experiments, the development of stable roof structure and surrounding rock cracks under repeated mining of close coal seams are further explored. And based on this, establish a coal face failure mechanics model to comprehensively analyze the influence of multiple roof structural instabilities on the stability of the coal face. Finally, numerical simulation is used to further supplement and verify the completeness and rationality of similar simulation experiment and theoretical analysis results. The results show that: affected by repeated mining disturbances, the cracks in the coal face are relatively developed, the strength of the coal body is reduced, and the coal face is more prone to failure under the same roof pressure; During the mining of coal seam 17#, the roofs of different layers above the stope form two kinds of "arch" structures and one kind of “voussoir beam” structure, and there are three different degrees of frequent roof pressure phenomenon, which is easy to cause coal face failure; Under repeated mining of close coal seams, the roof pressure acting on the coal face is not large. The main controlling factor of coal face failure is the strength of the coal body, and the form of coal face failure is mostly the shear failure of soft coal. The research results can provide a theoretical basis for coal face failure under similar conditions.

## Introduction

The occurrence conditions of coal seams in Guizhou province are mostly complicated with the characteristics of thinner thickness, larger gas bearing capacity, relatively soft roof and floor. Therefore, there are quite a few coal mines in Guizhou province mining the close "Three Soft" coal seams. It is easy to cause roof collapse and coal face failure under the repeated mining in close coal seams, which seriously restrict the safe and efficient mining of close coal seams^1–7^. It is of great theoretical and practical significance to the safe and efficient mining of the close coal seams group in Guizhou mining area to study the stability control of coal face under the repeated mining in the close coal seams group.

The attention to coal face stability began with the popularization and application of fully-mechanized mining face with large cutting height in China. It has been found that coal face stability is not only related to the strength and fault structure, but also to the advancing speed of the working face^8–10^. It is often effective to slow down the coal face failure by improving the advancing speed of working face^11–13^. However, it is limited to prevent the failure of coal face simply by speeding up the advancing speed of the working face with the increasing of the mining strength and the more and more complex mining conditions. Therefore, researchers gradually explore the influence factors, internal causes and reinforcement technology of coal face failure. The mechanism of the failure and prevention of coal face in extremely soft coal seam is analyzed^14–17^. It is considered that the coal face mainly shows two failure forms: fracture failure and shear failure. And reducing the pressure of coal face and improving the shear strength of coal body is the main controlling technical way. Regarding the problem of rib spalling in working face, scholars have done more research on its mechanical mechanism and control technology, obtained rich research results, and solved some problems of coal face failure^18,19^. Some researcher has simulated the development and evolution of mining cracks of coal seam, and applied the slip line theory to analysis of the failure mechanism of coal face, and got the dangerous scope of coal face failure^20^. The deflection of intact medium and hard coal were studied by means of pressure bar theory, and compared with the measured results in situ^21^. Some scholars designed a physical–mechanical model of a coal face that underwent shear failure and sliding instability in order to simulate the coal face failure process of coal mining under the overlying protective layer, and comprehensively analyzed the mechanism of coal rib spalling under the protective layer^22,23^. A mechanical model was established based on the interaction between the coal face, support and roof, and an expression describing the strength of the support and the rib spalling was proposed. The field experiment demonstrates that the long-hole hydrostatic pre-injection can control rib spalling and ensure the stability of the surrounding rock^24^. In addition, based on the "coal face-support-roof" mechanical model, it also shows the factors that affect the stability of the coal face, combined with numerical simulation to reveal the mechanical mechanism of coal face failure, and proposed the “manila + grouting” reinforcement technology to control the rib spalling^25^. A systematic study of the interaction mechanism between the support and the coal face shows that the equivalent resistance of the support has a negative linear relationship with the coal face displacement, and the relationship between the force of the support fender and the depth of the rib spalling is a hyperbolic function^26^.

On the basis of the above research, it is found that the research on the coal face failure and its control mechanism has played an important role in revealing the essence and basic law of coal face failure and roof caving and helping people to recognize the mechanism of coal face failure and the roof falls in unsupported areas. However, most of the above researches are focused on single seam mining, and there are few studies on the failure mechanism of coal face under repeated mining. The roof structure of the repeated mining is different from the single coal seam mining, and the stress environment of the coal face is also very different^27–32^. Therefore, it is necessary to study the stability of coal face under repeated mining in close distance coal seams. In the previous research, the authors have clarified the migration law of the overlying strata in close coal seams group mining, and on this basis, determined the reasonable working resistance of the working face support^33^. This paper is a further in-depth study on the basis of previous research, comprehensively using indoor testing, theoretical analysis, physical similarity simulation and numerical simulation methods to study the stability of the coal face under repeated mining in close coal seams.

## Engineering survey

The mine is located in the mining area of Pan Jiang in Guizhou. The geological structures are complex with widely distributed faults and folds. The mineable coal seams are 15#, 16#, 17# and 18#. The average thickness was 2.5 m, 2.0 m, 4.0 m and 5.0 m respectively, of which the interval between coal seam 15# and 16# is 6 m, the interval between coal seam 16# and 17# is 4–8 m, and the interval between coal seam 17# and 18# is 15 m. Therefore, the coal seam mining in this mine belongs to the mining of close-distance coal seams group. At present, coal seams 15# and 16# have been mined and coal seam 17# is being mined^33^. 17,101 is the first working face of coal seam 17#, and the mining method is comprehensive mechanized backward mining of large mining height method. The length of working face is 150 m, and the propelling length is 1000 m. The roof and floor strata of the mine are mostly siltstone and argillaceous siltstone, the cracks are more developed, and the roof is easy to caving.

The coal face failure and the roof caving are serious during the mining process of the working face 17,101, which restricts the safe, efficient and fast mining of the working face.

## Deformation and failure test of coal samples

The coal face failure in working face is related to the stress environment in the working face. From the setup entry to the first weighting or during the two periodic weighting, the stress environment of coal face changes from "three-dimensional" stress state to "two-dimensional" stress state and even "unidirectional" stress state. Therefore, the "unidirectional" stress state is the most unfavorable stress environment of coal face. In order to obtain the deformation and failure characteristics of coal samples under uniaxial compression, mechanics tests of coal samples of working face 17,101 were carried out.

**Figure 1:**
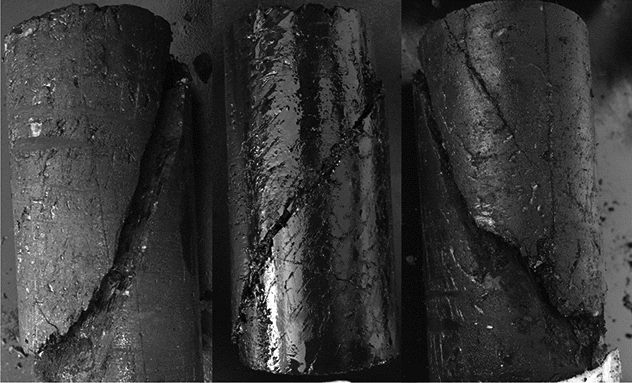
Failure characteristics of coal samples.

**Figure 2:**
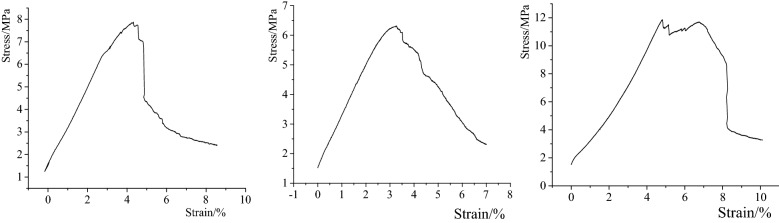
Stress–strain curve of coal samples.

The characteristics of coal samples after destruction are shown in Fig. 1. The stress–strain curves of coal samples are shown in Fig. 2.

As can be seen from Figs. 1, 2.

1. The failure form of coal samples under uniaxial compression is mainly shear failure, which runs through the bottom directly from the top, and the whole height is failure. There are many secondary cracks around the main failure surface, and secondary cracks will expand and develop further in the process of samples failure.
2. The uniaxial compressive strength of the samples is 6.5– 11 MPa, and the strain at the time of failure is 3.5– 7%, which shows plastic failure, or between brittle and plastic failure, and the residual strength is small.

From the above analysis, it is known that with the influence of repeated mining disturbance, the coal body cracks of coal face is more developed, the coal body strength becomes lower, the coal face is more prone to failure under the same roof pressure, and the failure form is mainly the shear failure of soft coal seams.

## Roof structure characteristics under repeated mining

The roof structure of repeated mining under close coal seams is far different from that of single stope coal seam. It is premise to study the activity rules and failure characteristics of roof and floor after the mining of coal seams 15# and 16# so as to obtain the roof structure characteristics of working face 17,101. In previous studies, it was found that the roof pressure of 17# coal seam is not strong in the mining of close distance coal seams, but in the rock layers of different layers, there are often three different degrees of roof pressure, and the coal seam is also easy to be failure^33^. Based on this, the physical similarity simulation experiment is used to further obtain the roof fracture structure of coal mining in different layers.

### Similarity simulation experiment scheme and model laying

To study the stability of coal face in working face 17,101, it is necessary to analyze the roof structure of the stope. However, with the continuous mining of close coal seams from top to bottom, the stable structure formed by the stope roof is also constantly changing. After repeated mining, the instability of the roof structure will inevitably have a significant impact on the stability of the coal face. Therefore, by constructing this physical similarity simulation experiment, by simulating the mining of coal seams 15#, 16# and 17# respectively, the stable roof structure formed by the actual close coal seams mining on site is restored to the greatest extent. Based on this, the stability of the coal face of the lower coal seam under the influence of repeated mining is analyzed.

**Figure 3:**
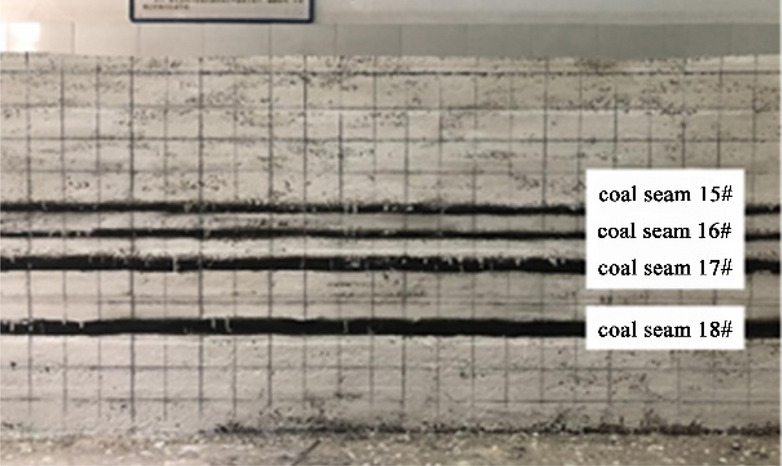
Similar simulation model.

The two-dimensional similar simulation test frame in this experiment has a length of 3.0 m, a width of 0.3 m and a height of 2.0 m. The periphery of the model and the bottom plate can be strongly restrained by the steel channel of the similar simulation test frame. The matching ratio of similar materials in each rock formation in this physical simulation experiment is the best result obtained after many experiments, as shown in Table 1. In addition, the material ratio of this experiment has been greatly changed. In order to ensure that the lithology of the simulated rock formation is more suitable, the actual situation is restored to a great extent, and the failure characteristics are better. Simulate different lithology materials with different matching ratios, using gypsum and lime as cementing materials, sand as aggregates. Due to the influence of repeated mining, the strength of the coal and rock masses is reduced, and the matching number of the model and the lime gypsum materials are appropriately reduced. For example, the matching number "746" for Fine sandstone, in which sand, lime and gypsum are distributed according to the weight ratio of 7:0.4:0.6. Under this ratio number, firstly, sand accounts for 7/8 of the total materials, and lime and gypsum together account for 1/8. Secondly, lime and gypsum are distributed according to 4:6. The model is laid according to the geometric similarity ratio 1:100, the bulk density similarity ratio 1:1.6 and the time similarity ratio 1:10^31^. The final laying model height is 1.2 m, in order to facilitate excavation and observation, a mesh format of 10 × 10 cm is arranged on the model (see Fig. 3). In this model, a total of 4 coal seams have been laid, among which coal seams 15#, 16#, and 17# belong to close-distance coal seams. After the model is laid, the upper coal seams (15#, 16#) are mined in sequence to simulate the upper goaf. Then the coal seam 17# was mined to observe the fracture characteristics of the roof and the stability of coal face of the coal seam 17# after being affected by repeated mining.

**Table 1:** Experimental material ratio table.

Lithology	Thickness (cm)	Matching number	Sand	Lime	Gypsum	Ratio of sand to lime gypsum	Ratio of lime to gypsum
Fine sandstone	20.00	746	7	0.4	0.6	7:1	4:6
Siltstone	20.00	846	8	0.4	0.6	8:1	4:6
Mudstone	10.00	828	8	0.2	0.8	8:1	2:8
Coal seam 15#	2.50	928	9	0.2	0.8	9:1	2:8
Mudstone	2.00	828	8	0.2	0.8	8:1	2:8
Fine sandstone	4.00	746	7	0.4	0.6	7:1	4:6
Coal seam 16#	2.00	928	9	0.2	0.8	9:1	2:8
Siltstone	6.00	846	8	0.4	0.6	8:1	4:6
Coal seam 17#	4.00	928	9	0.2	0.8	9:1	2:8
Fine sandstone	15.00	746	7	0.4	0.6	7:1	4:6
Coal seam 18#	5.00	928	9	0.2	0.8	9:1	2:8
Mudstone	10.00	828	8	0.2	0.8	8:1	2:8
Medium sand	20.00	737	7	0.3	0.7	7:1	3:7

### The roof structure characteristics after the mining of coal seam 15#

**Figure 4:**
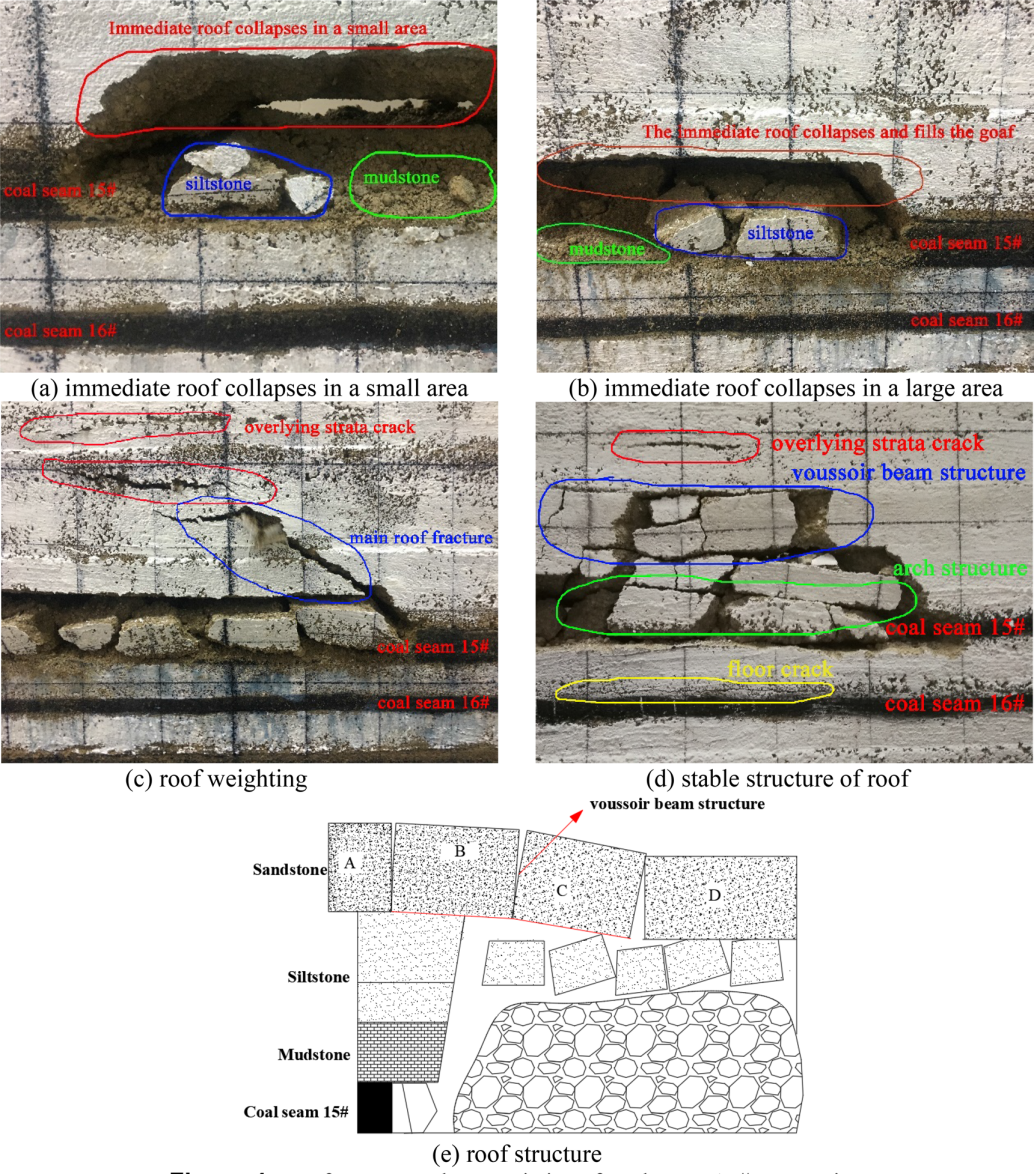
Roof structure characteristics of coal seam 15# excavation.

As shown in Fig. 4a, when the working face of coal seam 15# advances for about 30 m, the immediate roof collapses locally, and it is basically mudstone roof, while the siltstone roof only partially collapses; As shown in Fig. 4b, when the working face advances 40 m, the immediate roof collapses in a large area, and at this time the immediate roof collapse is mostly siltstone roof; As shown in Fig. 4c, when the working face continues to advance, the main roof fractures, the working face weighting, and more cracks are generated in the overlying strata; As shown in Fig. 4d, it is the roof structure formed after the main roof weighting is stabilized. It can be clearly seen that the main roof forms a stable "voussoir beam" structure, and the immediate roof also forms an "arch" structure to maintain stability. At the same time, affected by the first mining, the coal seam 15# floor rock and the overlying rock have different degrees of cracks, which will directly lead to the incomplete roof of the coal seam 16#. According to the analysis, the occurrence state of roof rock stratum after the mining of 15# coal seam is obtained as shown in Fig. 4e.

### The roof structure characteristics after the mining of coal seam 16#

**Figure 5:**
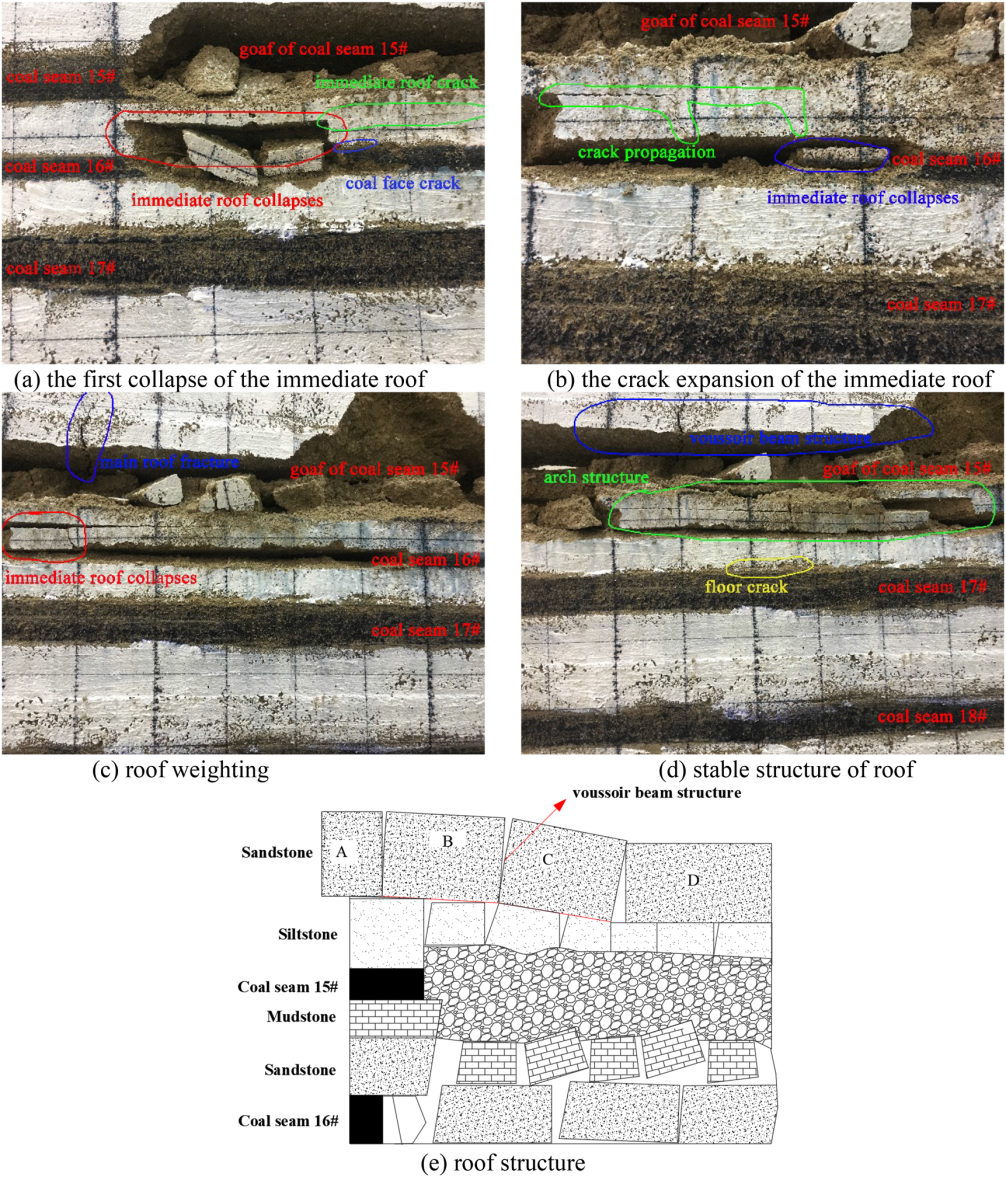
Roof structure characteristics of coal seam 16# excavation.

As shown in Fig. 5a, when the working face of coal seam 16# advances 20 m, the first collapse of the immediate roof, and all the goaf areas are filled, and at the same time large cracks appear in the immediate roof in front of the working face; As shown in Fig. 5b, when the working face advances 45 m, the immediate roof collapses in a large area, and the roof cracks continue to expand to the front of the working face; As shown in Fig. 5c, as the working face continues to advance, the immediate roof continues to collapse and cracks are generated to expand to the front of the working face. At the same time, the main roof of the coal seam 15# is fracture, and the stable "voussoir beam" structure and "arch" structure will be failure, which will cause impact and pressure on the roof of the coal seam 16#; As shown in Fig. 5d, it is the stable roof structure formed after the main roof pressure, the stable "voussoir beam" structure formed by the main roof after the second instability and the "arch" structure formed by the immediate roof can be clearly seen in the figure. Similarly, mining of the coal seam 16# will also cause cracks in the floor, which will affect the completeness of the roof of the coal seam 17#. According to this analysis, the occurrence state of the roof strata after the mining of coal seam 16# is shown in Fig. 5e.

### The roof structure characteristics after the mining of coal seam 17#

**Figure 6:**
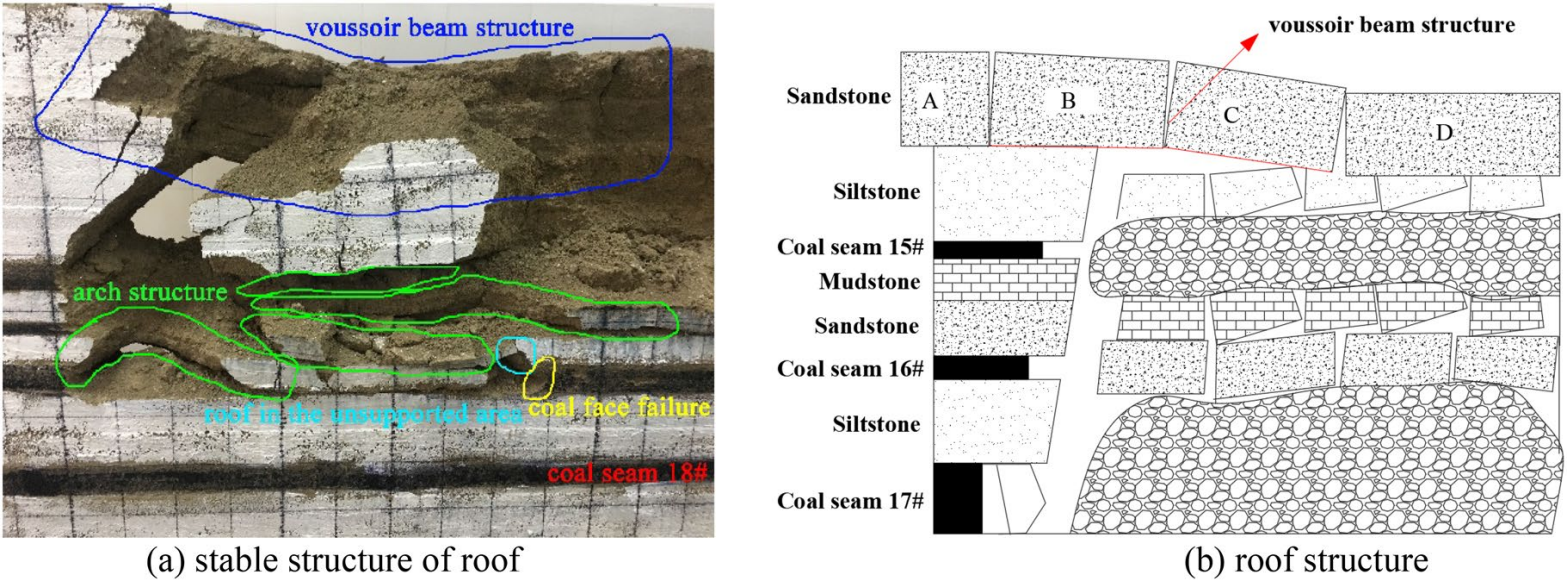
Roof structure characteristics of coal seam 17# excavation.

As shown in Fig. 6a, the mining of the coal seam 17# is carried out on the basis of the completion of the mining of the coal seams 15# and 16#, and the overlying strata failure is quite serious, but it can also be clearly seen that the stable "voussoir beam" structure formed by the main roof above the coal seam 15#, and the multi-layer "arch" structure formed above the goaf of the coal seam 17#. According to the analysis, the occurrence state of roof strata after the mining of coal seam 17# is obtained as shown in Fig. 6b. In addition, during the forward advancement process of the coal seam 17#, it was impacted by the instability of the "voussoir beam" structure and the "arch" structure for many times. It can be clearly seen that the coal face in front of the working face has been greatly failure. At the same time, the roof in the unsupported area is relatively broken and the stability is extremely poor, and further induces coal face failure.

## Force analysis of coal face under repeated mining

**Figure 7:**
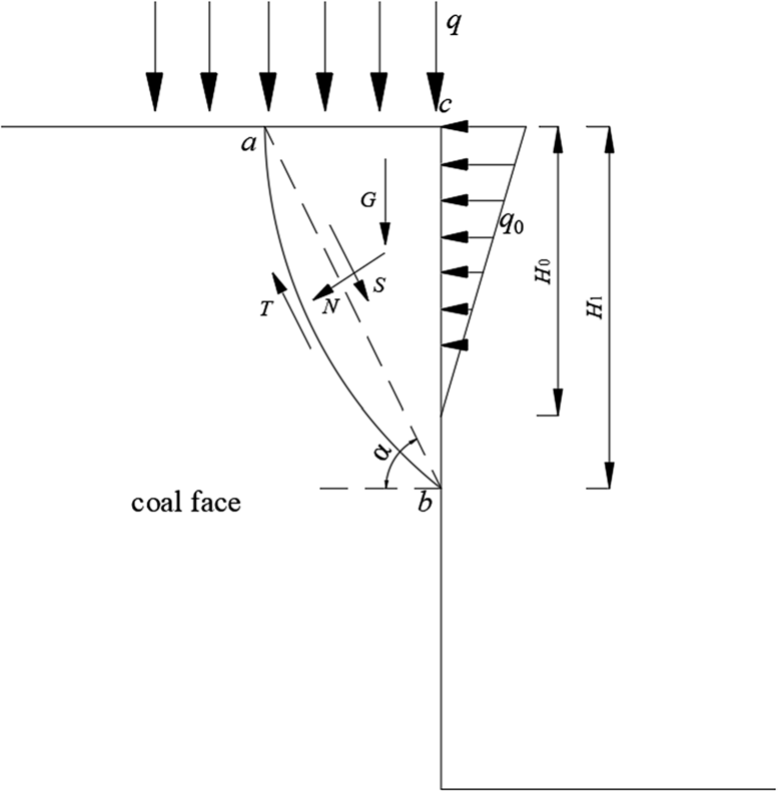
Mechanical model of coal face failure.

The coal face is more susceptible to failure under repeated mining of close-distance coal seam groups, and the most important influencing factor of coal face failure is the roof pressure on the coal face. According to the structural characteristics of the roofs of the various layers under repeated mining, it can be seen that the lower coal seams are more affected by the pressure of different layers of the overlying rock during the mining process, and the coal face is subject to more dynamic loads. From the compression phenomenon of roofs of different horizons combined with the shear face failure mechanical model of coal face^34,35^ is shown in Fig. 7. The stress analysis of the coal face of the coal seam 17# is as follows:

In the picture: *abc* is the range of coal face failure slip body; *q* is the pressure load on the coal face when the roofs of different layers are pressed; *q*_0_ is the load on the coal face by the support plate; *H*_0_ is the support height of the support plate; *H*_1_ is the coal face height of spalling; *G* is the self-weight of the failure slip body; *α* is the shear slip angle of the coal face failure; *T* is the shear force on the slip surface; *N* is the compressive stress on the slip surface; *S* is the failure surface sliding force. The coal face stability coefficient *K* is used to judge whether the coal face is failure.

From Mohr–Coulomb strength theory are:

1 $$ K = \frac{T}{S} = \frac{{\left[ {\left( {qH_{1} \frac{1}{\tan \alpha } + G} \right)\cos \alpha + q_{0} \frac{{H_{0} }}{2}\sin \alpha } \right]\tan \phi + cH_{1} \csc \alpha }}{{\left( {qH_{1} \frac{1}{\tan \alpha } + G} \right)\sin \alpha - q_{0} \frac{{H_{0} }}{2}\cos \alpha }} $$

where: $\phi$ is the internal friction angle of the coal seam 17#; *c* is the cohesion of the coal seam 17#; among them:

$$ G = \frac{1}{2}H_{1}^{2} \gamma \frac{1}{{{\text{tan}}\alpha }} $$

where: *γ* is the bulk force of coal. The basic data of this working face are: *γ* = 13 kN/m^3^, *H*_0_ = 2 m, *c* = 0.5 MPa,$\phi$ = 25°, *q*_0_ = 0.1 MPa, $\alpha = 45^{ \circ } + \phi /2 = 57.5^{^\circ }$.

Based on the “weighting pressure” phenomenon of different layers in the overlying strata and when there is no “weighting pressure”, the load *q* on the roof of the coal face is analyzed and calculated according to formula (2).

2 $$ \left\{ {\begin{array}{*{20}l} {P_{1} = \frac{{q_{1} B_{0} hL}}{h + (L - a)\mu }} \hfill \\ {P + Q = P_{1} + Q_{1} } \hfill \\ \end{array} } \right. $$

where *P*_1_ is the pressure on the lower layer when the upper rock layer forms a stable structure; *q*_1_ is the load concentration of the upper layer of the stable structure and its overlying roof; *B*_0_ is the width of the support: *h* is the thickness of the upper layer of the stable structure; *L* is weighting interval; *a* is the overhang length of the lower rock stratum of the stable structure; *μ* is the friction factor and the value is 0.7–1; *Q* is the roof pressure on the coal face; *Q*_1_ is the gravity of the immediate roof of the coal seam 17#; *P* is the roof pressure on the support.

1. The "arch" structure and the "voussoir beam" structure on the coal seam 17# are both in a stable state. At this time, the roof did not show the phenomenon of weighting pressure. The roof load *q* on the coal face was the smallest, and it was calculated that *q* = 1.58 MPa.
2. The "voussoir beam" structure formed by fine sandstone is unstable. When the structure is destabilized, although there is no dynamic impact, the gravity of rock block A and rock block B will all act on the coal face and support. At this time, the overburden rock load on the coal face will increase, and it is calculated that *q* = 1.92 MPa.
3. The "arch" structure formed by coal seam 15# siltstone roof or coal seam 16# fine sandstone roof is unstable, and the "voussoir beam" structure is stable. At this time, the coal face and the support will be strongly impacted, and at the same time, the gravity of the suspended rock blocks on the roof of the coal seam will all act on the coal face and the support. In this process, the two forces are superimposed, and *q* = 1.88 MPa is calculated.
4. The "arch" structure formed by the coal seam 15# siltstone roof or the coal seam 16# fine sandstone roof is unstable, and causes the structure of the overlying fine sandstone "voussoir beam" to be unstable. The increased force will superimpose on the coal face, and it is calculated that *q* = 2.3 MPa.
5. The "arch" structure and "voussoir beam" structure on the coal seam 17# are unstable at the same time. In this case, the coal face and support will be subjected to increased forces including the gravity of rock blocks A and B after the instability of the "voussoir beam" structure, the impact load and overhang caused by the instability of the "arch" structure of the overlying roof Gravity of rock blocks. The superposition of all forces is bound to cause the roof pressure on the coal seam 17# and coal face to be greater and it should be the maximum pressure at this time. The calculated *q* = 2.57 MPa.

The above several cases are the loads generated when the different layers of the lower coal seam are subjected to compression, and the analysis is relatively independent. During the normal mining of the coal seam, the cycles of the different layers of the overlying rock layer are accompanied, periodic weighting may be asynchronous, so the overburden roof load that the coal wall is subjected to is also different in different periods. Therefore, when mining lower coal seams in close distance coal seam groups, "weighting pressure" is more frequent than single coal seam mining. Since the first weighting, the coal face will be repeatedly subjected to roof loads of different sizes every time the front roof is pushed forward with the working face, which is extremely detrimental to the stability of the coal face.

**Figure 8:**
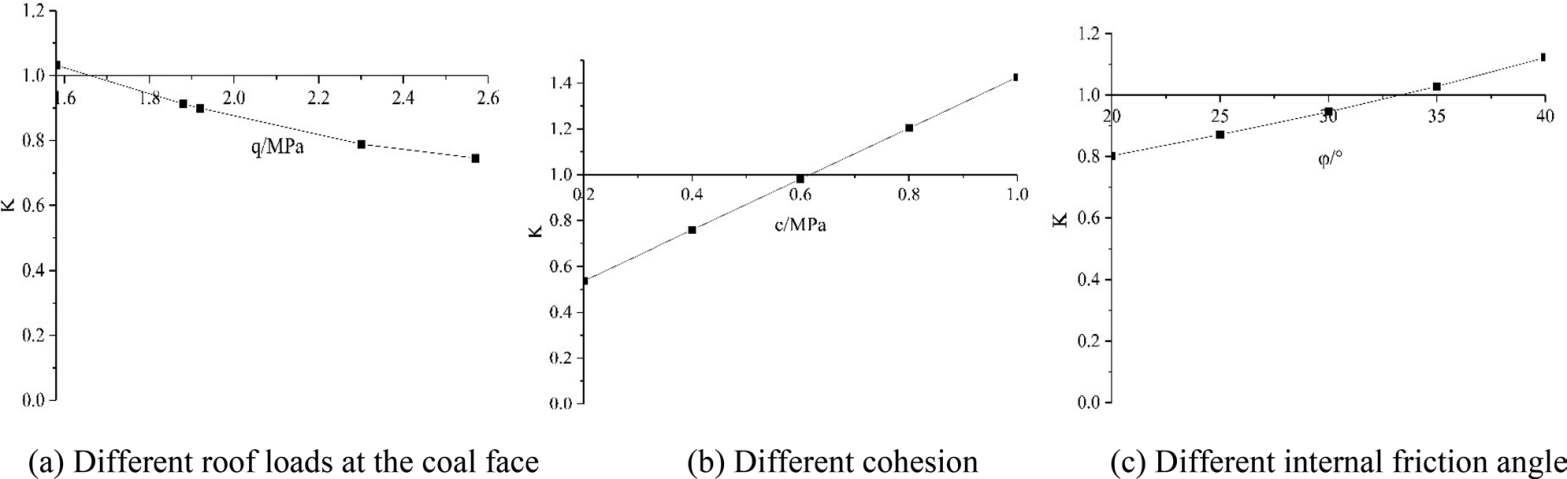
Coal face stability curve.

From the above analysis and calculation, according to formula (1), the stability coefficient curve of the coal face under different roof loads (*q*) and different coal body characteristics (*c*, *φ*) can be obtained as shown in Fig. 8.

As shown in the Fig. 8a, when the coal seam 17# is being mined, when the overlying stratum structure remains stable, the coal face under the small roof load will remain stable. At this time, it should be not far from the front of the setup entry of the working face and no first weighting. With the continuous advancement of the working face, the roof pressure on the coal face continues to increase. When the coal face roof pressure reaches about 1.65 MPa, the coal face stability coefficient K value is less than 1 and the failure will start; When the "arch" structure or "voussoir beam" structure is unstable, the coal face will be failure to a large extent.

As shown in Fig. 8b and c, under a certain roof pressure at the coal face (in the process of instability of the "arch" structure and "voussoir beam" structure, the roof pressure is about 2 MPa), with the increase of cohesion and internal friction angle in coal body, the stability coefficient K of coal face also increases, and the stability of coal face is better. As shown in Fig. 8b, under this roof pressure, when the coal body cohesion is less than about 0.62 MPa, the coal body strength is low, and the coal face stability coefficient K is less than 1, and the coal face will be spalling; As shown in Fig. 8c, under this roof pressure, when the internal friction angle in the coal body is less than about 33°, the strength of the coal body is low, and the coal face stability coefficient K is less than 1, and the coal face will be spalling.

Affected by repeated mining disturbances, on the one hand, the cracks in the coal face are relatively developed, and the strength of the coal body becomes lower (cohesion and internal friction angle are reduced), and the coal face is more prone to failure at the same roof pressure; On the other hand, with the cyclic instability of the "arch" structure and the "voussoir beam" structure, the roof pressure at the coal face changes constantly and increases at any time, which is extremely detrimental to the stability of the coal face.

## Numerical simulation of coal face failure under repeated mining

### Establishment of numerical model of close coal seams group

In order to obtain the coal face failure condition in a large-cutting-height panel under repeated mining, the UDEC software is used to simulate the coal face failure under the background of the geological and mining conditions of the close coal seams in a mine.

The plane strain model is employed to simplify the calculation. The UDEC model is 100 m in length and 120 m in height, with two 20 m protective coal pillars on both sides^36^.

**Figure 9:**
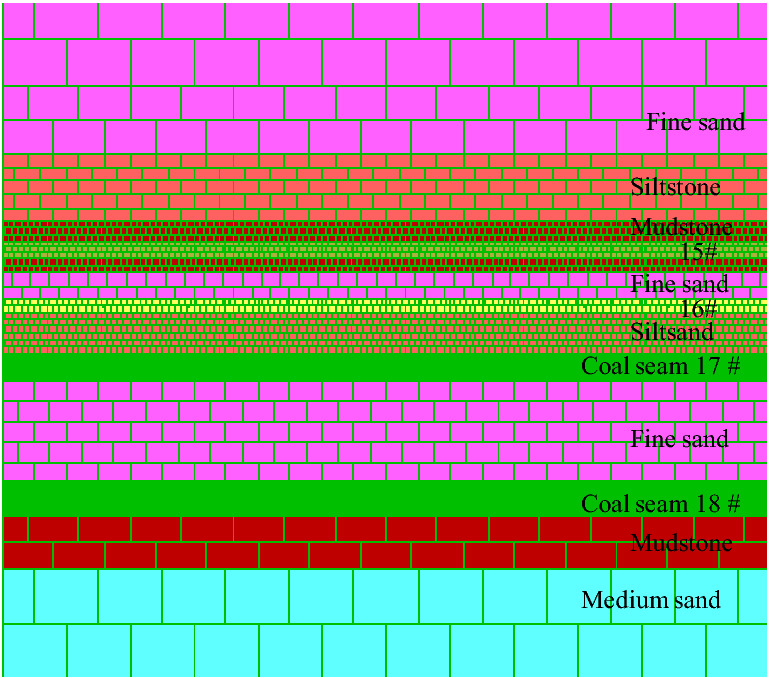
Numerical simulation model.

A uniform vertical stress of 12.5 MPa is applied on the upper model boundary. Roller boundaries are used for both sides in the Y direction and for the bottom^36^. In the numerical model, the unsimulated rock formation above the model is replaced by the equivalent load. The numerical model is shown in Fig. 9. The mechanical parameters of coal and rock mass are shown in Table 2, and the physical and mechanical parameters of joints are shown in Table 3.

**Table 2:** Mechanical parameters of coal and rock.

Lithology	Density (kg/m^3^)	Cohesion (MPa)	Friction (°)	Bulk (GPa)	Shear (GPa)	Tension (MPa)
Medium sand	2500	5.9	42	7.38	6.96	4.56
Siltstone sand	2540	5.2	40	6.85	5.47	3.86
Fine sandstone	2600	4.38	39	5.27	4.69	3.35
Mudstone	2550	1.24	37	4.16	2.83	3.02
Coal seam	1350	0.5	30	3.95	2.2	1.04

**Table 3:** Joints mechanical parameters of coal and rock mass^33^.

Lithology	Jkn (MPa)	Jks (MPa)	Jfri (°)	Jcoh (MPa)	Jten (MPa)
Medium sand	5500	5960	22	2.38	1.56
Siltstone sand	4540	4800	19	1.85	0.86
Fine sandstone	3600	4380	18	1.27	0.75
Mudstone	2550	2240	17	0.86	0.22
Coal seam	2350	2320	15	0.45	0.04

During the excavation process of the numerical model, the roof failure and the stability of the coal face at the working face of the coal seam 17# were analyzed from the following three mining schemes. That is, the coal seams 15# and 16# are not mined and only the coal seam 17# is mined separately, the coal seam 15# has been mined and the coal seam 16# is not mined, and the coal seams 15# and 16# have been mined and then the coal seam 17# has been mined.

### Analysis of simulation results of mining in different coal seams

#### Single mining of coal seam 17#

**Figure 10:**
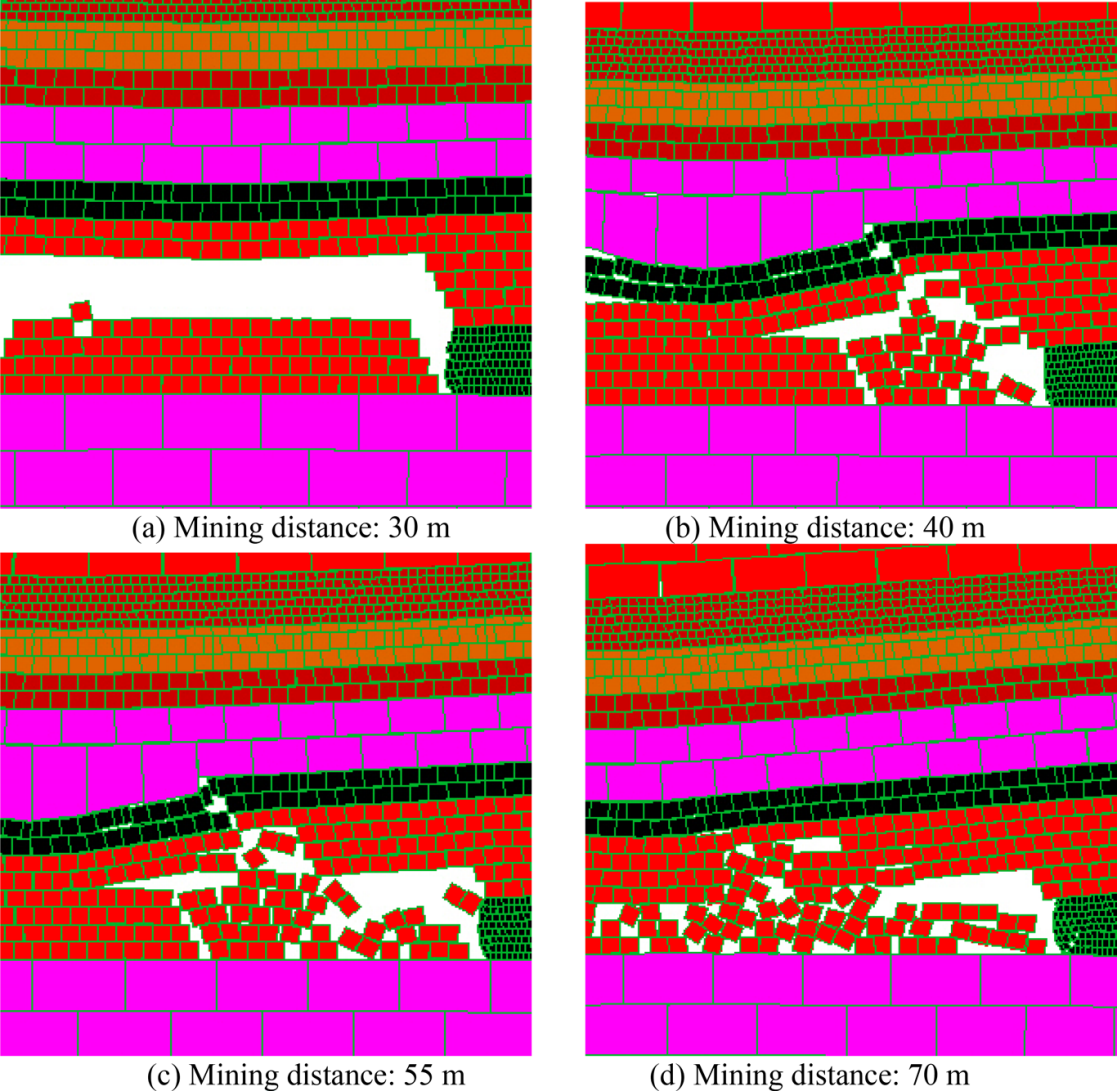
The coal face stability at the mining conditions of coal seams 15# and 16# are not mined.

When only coal seam 17# is mined, the stability of the working face under different mining distances is shown in Fig. 10:

As can be seen from Fig. 10, when the working face advances to 30 m, the immediate roof appears a wide range of collapsing but the coal face remains stable; When the mining distance is 40 m, the initial weighting of main roof occurs, and the coal face remains stable at this time; When the mining distance is 55 m, the first periodic weighting occurs, and the deformation of coal face increases, but it has not been destroyed; When the mining distance is 70 m, the small failure of coal face near the floor occurs, but the overall height of coal face remained stable. Therefore, there are no coal face accidents at the condition of single mining of only coal seam 17#.

#### The coal seam 15# has been mined and the coal seam 16# is not mined

**Figure 11:**
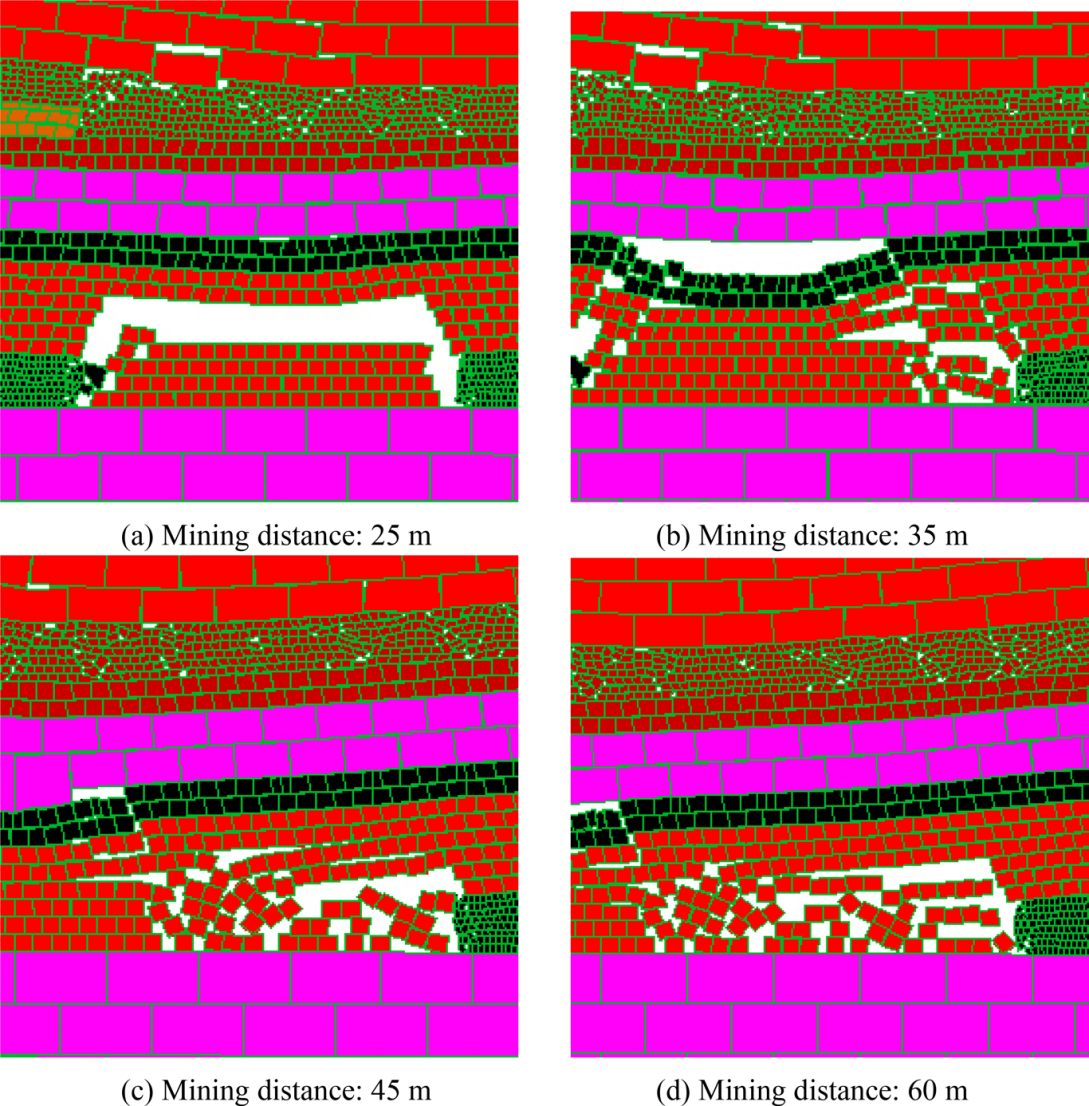
The coal face stability at the mining conditions of coal seam 15# has been mined.

When the coal seam 15# is mined and the coal seam 16# is not mined, affected by the coal seam 15# mining, the stability of the working face with different mining distance when the coal seam 17# is mined are shown in Fig. 11:

As can be seen from Fig. 11, influenced by the mining disturbance of coal seam 15#, when the working face mines to 25 m, the immediate roof appears a wide range of collapsing, although the coal face remains stable, the deformation is larger; When the mining distance is 35 m, the initial weighting of main roof occurs, and the coal face has small failure at this time; When the mining distance is 45 m, the first periodic weighting occurs, and the upper and middle part of the coal face has a certain degree of failure; When the mining distance is 60 m, the overall height of coal face shows a certain degree of coal face spalling, and the maximum failure depth is 0.9 m. Therefore, influenced by the mining of coal seam 15#, during the mining process of coal seam 17#, there will be some accidents in the coal face, but the coal face failure degree is not very serious.

#### The coal seams 15# and 16# have been mined

**Figure 12:**
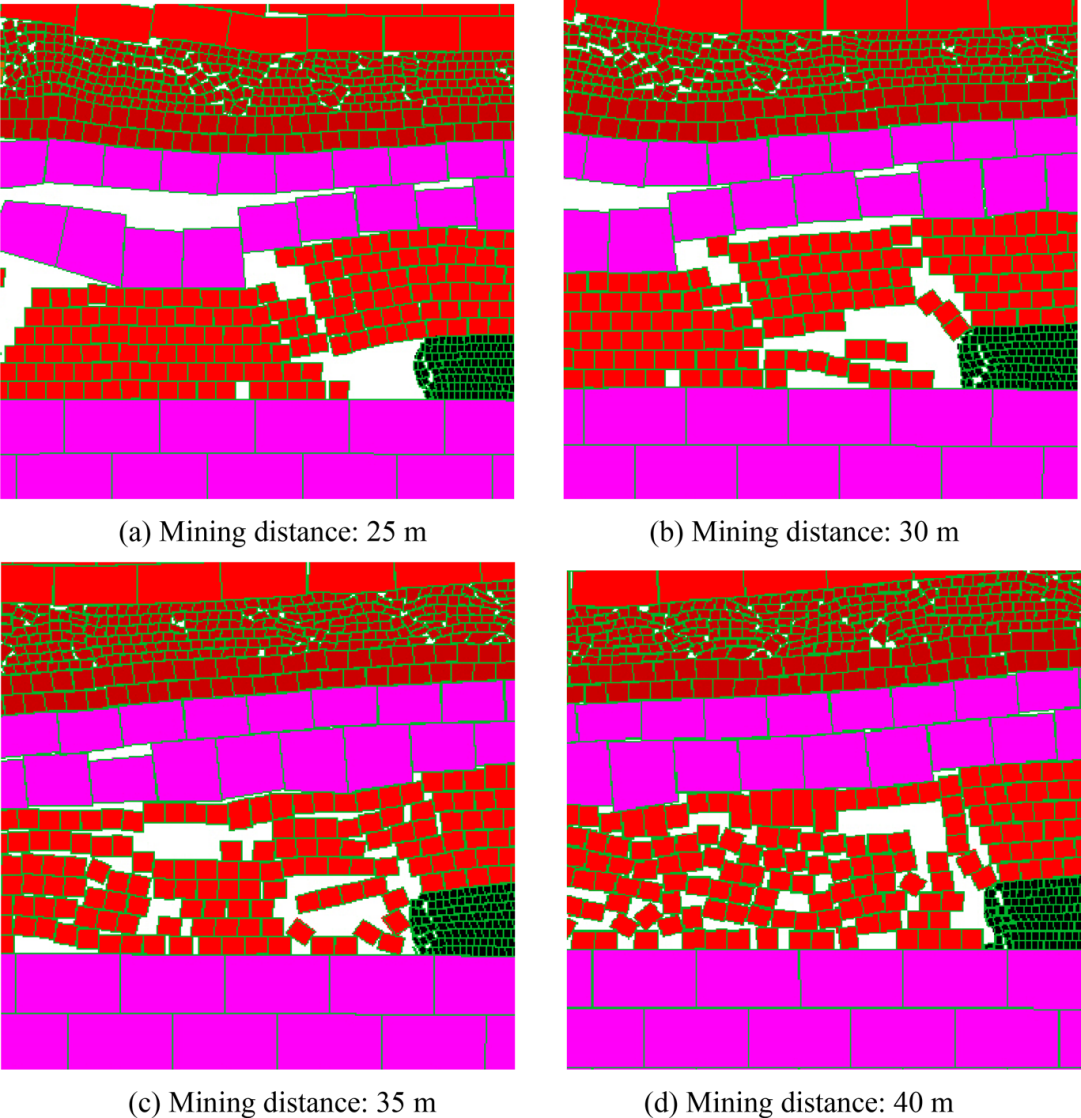
The coal face stability at the mining conditions of coal seams 15# and 16# have been mined.

When the coal seams 15# and 16# are mined and affected by the upper coal seam mining, the stability of the working face at different mining distances when the coal seam 17# is mined are shown in Fig. 12:

As can be seen from Fig. 12, influenced by the repeated mining disturbance of coal seams 15# and 16#, fissures of strata are very developed. When the working face mines to 25 m, a large scale caving occurred in the immediate roof, and the caving height and caving area are much larger than the previous two, and the coal face was failure and the maximum failure depth was 0.6 m; When the mining distance is 30 m, the initial weighting of main roof occurs, and the overall height of coal face is failure with the maximum failure depth of 1.5 m; When the mining distance is 35 m, the first periodic weighting occurs, and the overall height of coal face is also failure with the maximum failure depth of 1.2 m. With the mining of the working face, there is a roof weighting phenomenon every time the working face advances 5 m, and the coal face spalling and roof caving of end face will occur. Therefore, influenced by the repeated mining of coal seams 15# and 16#, due to the more frequent roof weighting and the more developed fissures, the coal face failure frequently occurs, and the failure degree is more serious, showing the shear failure on the whole height.

## Conclusions

Aiming at the problem of easy instability and failure of coal face in close coal seams mining, a variety of research methods are used comprehensively, and coal samples mechanics tests have made it clear that coal strength is one of the factor that affect coal face stability. Through physical similarity simulation experiment, we further explored the stable roof structure formed by repeated mining in close coal seams and the evolution characteristics of roof and floor cracks under the influence of mining. Based on this, the coal face failure mechanical model is constructed to analyze the influence of multiple roof structural instabilities on the stability of the coal face. In addition, the results of similar simulation experiment and theoretical analysis are further supplemented and verified by numerical simulation. The main conclusions are as follows:

1. Due to the influence of repeated mining of close coal seams, coal body cracks of coal face is more developed, the coal body strength becomes lower as well, and the coal face is more prone to failure at the same roof pressure.
2. During the mining of coal seam 17#, two kinds of "arch" structure and one kind of "voussoir beam" structure will be formed at the top of different strata above the stope, and there are three different degrees of roof weighting in the mining of working face. Frequent roof weighting is prone to cause rib spalling and roof caving, which is a major reason that coal face failure in the mining of close coal seams. On the other hand, due to repeated disturbance, the roof in the unsupported area is more broken, which easily leads to roof falls in the unsupported area, thus, the roof in the unsupported area increases the no support space, and further induces coal face failure.
3. The roof pressure on coal face is not large under the repeated mining of the coal seams group, the main controlling factor of coal face failure is the strength of coal body, and the coal face failure form is mostly the shear failure of the soft coal body. Under this condition, the prevention and control measures are to reinforce the coal wall and the end roof.

## Data Availability

All data and models or used during the study appear in the submitted article. And all data analysed during this study are included in this published article https://doi.org/10.1007/s10706-019-00879-0, and in S.S. Zheng, Y.H. Lou, D.Z. Kong, G.Y. Wu, Y. Liu, The Roof Breaking Characteristics and Overlying Strata Migration Law in Close Seams Group Under Repeated Mining.
